# Progress in Polysaccharide-Based Hydrogels for Preventing Postoperative Adhesions: A Review

**DOI:** 10.3390/gels11030188

**Published:** 2025-03-08

**Authors:** Mengyao Chen, Jialin Liu, Jianhong Lin, Kai Zhuang, Yudong Shan, Sandip Tiwari, Lei Jiang, Jiantao Zhang

**Affiliations:** 1 School of Materials Science and Chemical Engineering, Ningbo University, Ningbo 315211, China; 2 Laboratory of Advanced Theranostic Materials and Technology, Ningbo Institute of Materials Technology and Engineering, Chinese Academy of Sciences, Ningbo 315201, China; 3 Zhejiang Key Laboratory of Biopharmaceutical Contact Materials, Ningbo Institute of Materials Technology and Engineering, Chinese Academy of Sciences, Ningbo 315201, China; 4 Ningbo Cixi Institute of Biomedical Engineering, Cixi, Ningbo 315300, China; 5 University of Chinese Academy of Sciences, Beijing 100049, China; 6 Pharma Solutions, Nutrition and Health, BASF (China) Company, Ltd., 333 Jiang Xin Sha Road, Shanghai 200137, China; 7 Hangzhou Zhongmeihuadong Pharmaceutical Co., Ltd., 866 Moganshan Road, Hangzhou 310011, China; 8 Pharma Solutions, BASF Corp., 500 White Plains Rd, Tarrytown, NY 10591, USA

**Keywords:** polysaccharide, postoperative adhesions, hydrogels

## Abstract

Postoperative adhesions are common complications following surgery, often accompanied by pain and inflammation that significantly diminish patients’ quality of life. Moreover, managing postoperative adhesions incurs substantial cost, imposing a considerable financial burden on both patients and healthcare systems. Traditional anti-adhesion materials are confronted with limitations, such as inadequate tissue adherence in a moist environment and poor degradability, underscoring the urgent need for more effective solutions. Recently, polysaccharide-based hydrogels have received considerable attention for their potential in preventing postoperative adhesions. The hydrogels not only facilitate wound healing but also effectively reduce inflammation, providing a promising approach to preventing postoperative adhesions. This review provides an extensive analysis of the progress made in the development of polysaccharide-based hydrogels for postoperative anti-adhesion therapy. It highlights their principal benefits, outlines future research trajectories, and addresses the ongoing challenges that need to be overcome.

## 1. Introduction

Postoperative adhesion is a common and serious complication encountered across nearly all surgical disciplines. This complication can occur anywhere in the body, but is more common after surgeries on the peritoneum, pericardium, tendons, uterus, and epidural space [1,2]. As a result, patients may be at great risk for long-term complications such as chronic pain, bowel obstruction, infertility, and other complications [3]. Moreover, studies have shown that adhesions are frequently associated with significant morbidity and mortality, especially in patients who require repeated surgical interventions [4]. Furthermore, adhesions do not just affect patients, they also place a heavy burden on the economy and the healthcare system. In the United States, for example, the problem costs the healthcare system up to USD 2.5 billion annually [5]. At the same time, adhesions also complicate subsequent surgical procedures, requiring more time and resources from surgeons, which not only raises the risk of surgery but also increases the burden on healthcare workers.

**Figure 1:**
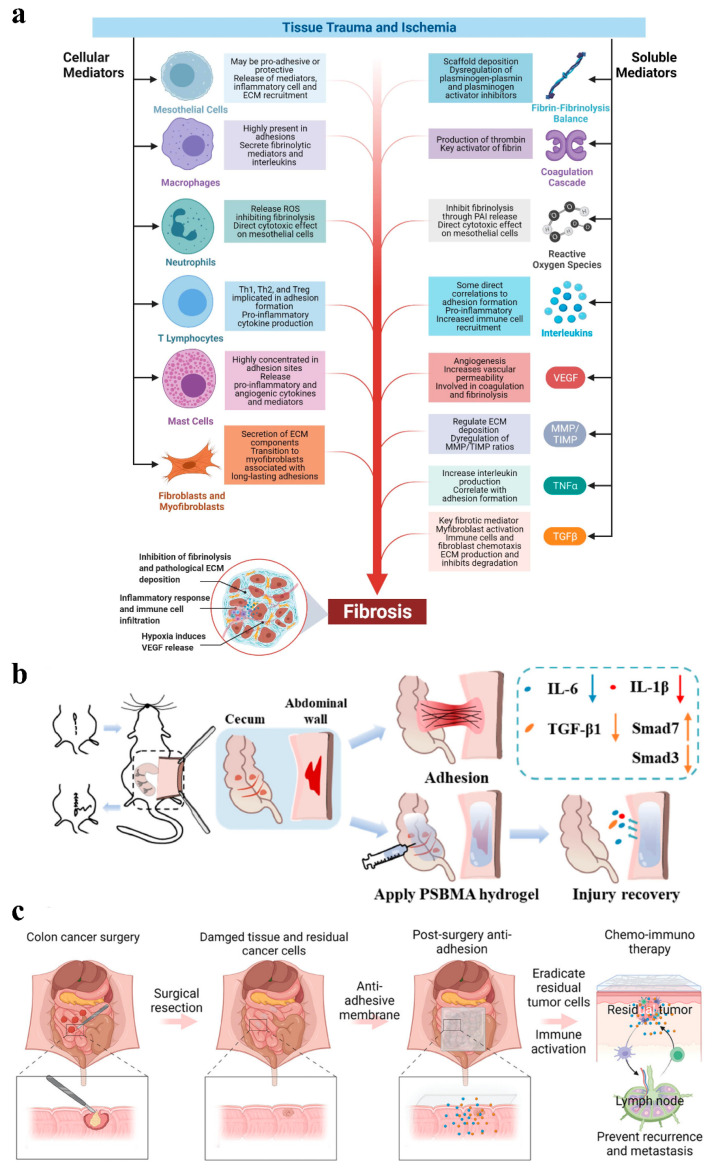
(**a**) Mechanisms of adhesion formation: (1) Inhibition of fibrinolysis and ECM deposition. In this scenario, fibrin deposits become challenging to eliminate, and the accumulation of the ECM surpasses the tissue repair requirements; (2) Inflammatory response. This involves the increased production of TGF-β1. By regulating ECM deposition, TGF-β1 facilitates the formation and progression of adhesions; (3) Hypoxia. It stimulates increased expression of VEGF and TGF-β1 and TGF-β2 and enhances angiogenesis, leading to excessive ECM deposition and hyperproliferation of fibrous tissue, ultimately inducing adhesion formation [4]; (**b**,**c**) Physical isolation: (**b**) The lateral wall model of the cecum was established and the different effects with and without hydrogel treatment. Among them, the group with hydrogel applied effectively inhibited fibrosis by decreasing the levels of IL-6, IL-1β and TGF-β1 and activating the TGF-β1/Smad7 and limiting the TGF-β1/Smad3 signaling pathway, whereas the group without hydrogel experienced adhesions [23]; (**c**) An electrostatic spinning membrane to prevent adhesion and inhibit tumor recurrence or metastasis by removing residual tumor cells and immune activation [22].

Previous studies defined adhesions as scar tissue between the internal organs and body walls [6]. However, with the advancement of research, the understanding of adhesions has evolved. Herrick et al. demonstrated that adhesions are highly cellular, vascularized, and innervated, resembling dynamic regenerative tissues rather than static fibrous scar tissue characterized by poor vascularization [7]. The formation of adhesions is driven by the combined action of various growth factors and is primarily due to an imbalance between fibrin deposition during coagulation and its breakdown through the fibrinolytic system [2]. The inflammatory state plays a crucial role in maintaining the balance between fibrin formation and fibrinolysis [8]. The mechanism of adhesion formation consists of three inductive processes (as shown in Figure 1a). The first process is a reduced capacity for fibrinolysis and extracellular matrix (ECM) degradation during surgery [9,10]. In this scenario, fibrin deposits become challenging to eliminate, and the accumulation of the ECM surpasses the tissue repair requirements. This creates a microenvironment for the development of postoperative adhesions, thereby increasing the risk of adhesion formation. The second process is the progression of the inflammatory response, which involves the increased production of transforming growth factor-β1 (TGF-β1), a key regulator of ECM deposition [11]. By regulating ECM deposition, TGF-β1 facilitates the formation and progression of adhesions. The third process is the onset of hypoxia, which stimulates increased expression of vascular endothelial growth factor (VEGF) and transforming growth factor β1 and β2 (TGF-β1 and TGF-β2) [12]. Hypoxia enhances angiogenesis [13], leading to excessive ECM deposition and the hyperproliferation of fibrous tissue, ultimately inducing adhesion formation.

Postoperative adhesions not only affect patient comfort, but may also hinder wound healing. Adhesions primarily develop when injured tissue comes into contact with adjacent healthy tissue. While laparoscopic surgery has been shown to reduce adhesion incidence compared to open surgery, both approaches still pose risks [14]. Therefore, it is essential to develop an effective wound closure strategy that minimizes tissue contact. Sutures, as a common wound closure technique, offer high tensile strength [15]. However, their use is not ideal due to the need for anesthesia during placement, and their effectiveness may vary depending on the physician’s expertise [16]. In contrast, staples are faster to apply and cause less pain during use [17], but they tend to be less comfortable when being removed [18]. Tapes can be applied more quickly and bring minimal discomfort to patients. However, they have low tensile strength and require the use of adhesive agents. This dependency increases the risk of inflammatory response and infection [19]. Additionally, these methods may create contact points between tissues, further exacerbating inflammation and adhesion formation. As a result, they are ineffective in reducing the occurrence of adhesions. To address this issue, two feasible preventive strategies have emerged to mitigate the risk of postoperative adhesions: (1) the use of drugs to reduce the inflammatory response [20,21], and (2) the application of physical barriers to isolate damaged tissue surfaces (Figure 1b,c) [22,23].

Although various drugs have been developed to prevent postoperative adhesions, their effectiveness is limited due to potential toxicity and inconsistent efficacy [20]. Therefore, physical isolation has become the most promising clinical strategy for preventing postoperative adhesions [24]. The use of polymer solutions, films, and hydrogels to separate injured tissues from adjacent healthy tissues has shown promise in effectively preventing adhesion formation. However, polymer solutions and films face challenges, such as inadequate adhesion and slow degradation rates [25]. Due to their excellent biocompatibility and biodegradability, hydrogels have gained growing interest as barriers for preventing postoperative adhesions. This has encouraged significant research and development efforts aimed at optimizing hydrogel-based solutions for clinical applications. Among the various types of hydrogels, polysaccharide-based hydrogels have emerged as a promising class of materials for preventing postoperative adhesions. Polysaccharides possess unique properties, such as biocompatibility, biodegradability, and tunable mechanical characteristics, which make them highly suitable for use in hydrogel barriers [26]. Polysaccharide-based polymers can be readily modified to tailor their mechanical properties, degradation rates, and biological interactions, thereby enhancing their efficacy in preventing adhesion formation. This article focuses on the classification, structure, and applications of polysaccharide-based hydrogels in postoperative anti-adhesion strategies.

## 2. Classification of Polysaccharide-Based Hydrogels

**Figure 2:**
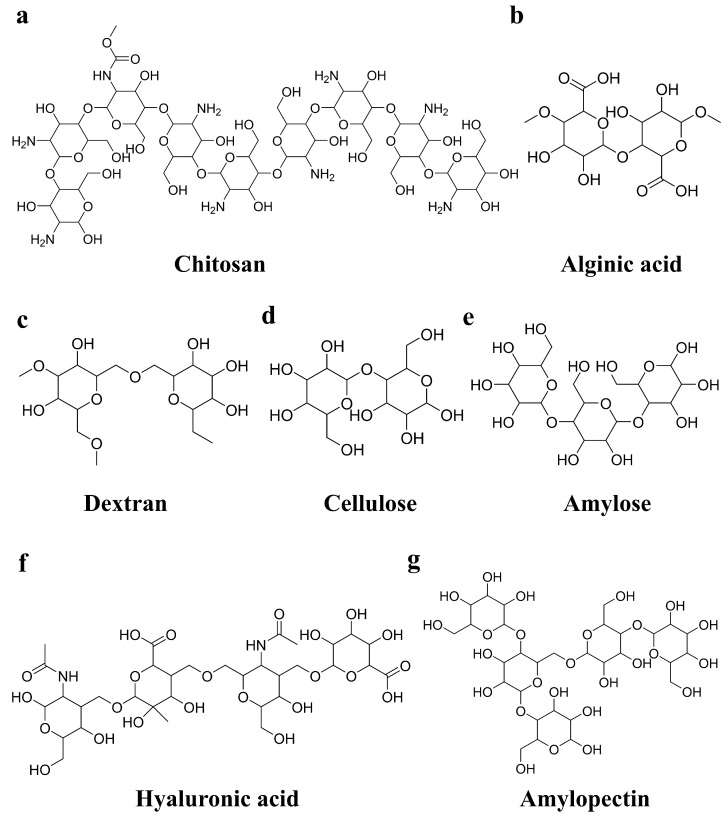
Structural formulas of selected polysaccharides: (**a**) chitosan; (**b**) alginic acid; (**c**) dextran; (**d**) cellulose; (**e**) amylose; (**f**) hyaluronic acid; (**g**) amylopectin.

As one of the most abundant natural polymers on earth, polysaccharides are widely found in animals, plants, algae, and microorganisms [27]. In recent years, polysaccharide-based materials such as chitosan [25,28], hyaluronic acid [29,30], cellulose [31], and pectin [32] have gained attention as promising candidates for postoperative anti-adhesion hydrogels. Their structures are illustrated in Figure 2. These materials are highly valued due to their good biocompatibility, biodegradability, and beneficial biological activities, including antibacterial and anti-inflammatory properties [26]. However, the relatively low mechanical strength of polysaccharide-based hydrogels makes it challenging to ensure effective adhesion prevention when they are used alone [33]. To address these limitations, modifications to polysaccharide materials are essential to improve their performance as part of anti-adhesion strategies. The following section provides a brief overview of the properties of several polysaccharide-based hydrogels and briefly summarized in Table 1.

### 2.1. Cellulose-Based Hydrogels

As the most abundant polysaccharide in nature [34], cellulose has garnered significant research interest due to its low cost, strong processability, good flexibility, biocompatibility, and biodegradability [35]. The presence of numerous hydrophilic functional groups in cellulose makes it an ideal candidate for hydrogel preparation [36]. Cellulose-based hydrogels have found promising applications in drug delivery, cell culture, biosensors, and drug development within the biomedical field [37]. However, the hydrogen bonds and van der Waals forces in cellulose limit its solubility in water and most organic solvents. Moreover, cellulose-based hydrogels lack inherent antimicrobial activity, posing a challenge for their broader application [38].

To overcome these limitations and enhance the potential of cellulose, physical and chemical modifications are commonly employed. These modifications alter the surface properties and functional groups of cellulose, endowing it with new characteristics suitable for various applications [39]. For example, a double-network conductive hydrogel composed of cellulose and gelatin has been developed. The results of this study showed that this hydrogel significantly improves resistance to swelling and possesses strong adhesion properties that are stable over time, properties that are precisely necessary for the manufacture of postoperative anti-adhesive hydrogels [40]. Moreover, the integration of antimicrobial materials like metal oxides into cellulose-based hydrogels has led to the creation of antibacterial hydrogels [41]. For example, George et al. prepared a nanocomposite hydrogel loaded with zinc oxide nanoparticles and curcumin. The results showed that this hydrogel exhibited synergistic antibacterial activity against *S. aureus* and *Trichophyton rubrum*. In addition, this nanocomposite hydrogel significantly increased drug loading, providing a new idea for combining postoperative anti-adhesion hydrogels with drug loading [42].

### 2.2. Chitosan-Based Hydrogels

Chitosan, a naturally occurring cationic polysaccharide polymer, stands out as the sole alkaline polysaccharide in nature [43,44]. It possesses a large number of active amino groups and hydroxyl groups, which can be modified through acylation, carboxylation, and etherification to obtain a series of chitosan derivatives with distinct properties, thereby expanding the utility of chitosan [45]. However, their limited mechanical strength poses a challenge, potentially hindering their effective application on the irregular surfaces of wounds and affecting the desired healing outcomes. However, the crosslinking and intermolecular interactions facilitated by chitosan can imbue hydrogels with self-healing capabilities, enabling them to adapt to the irregular shapes of wounds. For example, Song et al. prepared a cordycepin/chitosan hydrogel dressing (CY/CS) using a one-step “freeze-thaw” method. It has self-healing properties and can adapt to irregular wound surfaces. Meanwhile, its loaded cordycepin has antioxidant and anti-inflammatory effects. These are innovative for postoperative anti-adhesion applications [46]. Additionally, a strengthened chitosan-based hydrogel was prepared by combining dopamine with chitosan through the carboxyl function of citric acid, thereby overcoming limitations in mechanical properties. The results showed that this hydrogel significantly promoted cell regeneration at the injury site and reduced the inflammatory response. This novel modification strategy not only opens a new way for the structural modification of chitosan, but also brings new insights into the therapeutic strategy of spinal cord injury and the expansion of the application of chitosan [47].

### 2.3. Sodium Alginate-Based Hydrogels

Alginate is a natural anionic polysaccharide [48]. It is usually obtained from brown seaweed and is generally insoluble in water, but it can be completely dissolved in water after being combined with Na^+^. Furthermore, it is composed of blocks of alternating residues of α-L-guluronic (G) and β-D-mannuronic (M) [49]. Among them, the G block is related to the hardness of the hydrogel, while the M block affects the elasticity of the hydrogel [50]. It is widely accepted that only the G block of alginate is involved in intermolecular crosslinking with divalent cations, such as Ca^2+^, which leads to the formation of hydrogels with egg-box conformation. Hence, the primary factors that affect the physical properties of alginate and its resulting hydrogels include the composition (specifically, the M/G ratio), sequence distribution, and length of the G block and the molecular weight of the alginate [51,52]. Therefore, Wu’s team investigated the effect of different M/G ratios on hydrogel properties. The findings indicated that at an M/G ratio of 2:1, the anionic groups of calcium β-hydroxy-β-methylbutyrate (CaHMB), which include carboxyl and hydroxyl groups, formed hydrogen bonds with the polysaccharide chains. This interaction impeded the sequestration of Ca^2+^ by the G-block residue, thereby slowing down the gelation process. Moreover, the non-calcium crosslinked alginate and CaHMB’s anionic groups influenced hydrogel water distribution, particularly at higher M than G content. A decrease in the M/G ratio led to an increase in the number of “egg box” crosslinks in calcium alginate, resulting in a denser microstructure within the hydrogel pores, enhanced hydrogel strength, and increased binding of free water within the hydrogel matrix [53]. However, the degradation of pure alginate hydrogels in vivo is slow, which limits their application. Fortunately, sodium periodate (NaIO4) can oxidize the hydroxyl groups of alginate to aldehyde groups, providing more active sites and thus becoming biodegradable [54,55]. Hence, Zhao and colleagues meticulously devised an alginate oxidation strategy to produce hydrogels that exhibit more appropriate degradation rates. And they thoroughly investigated the physical and biological characteristics of these hydrogels both in vitro and in vivo [56].

### 2.4. Hyaluronic Acid-Based Hydrogels

Hyaluronic acid is a naturally occurring linear polysaccharide and an important component of the extracellular matrix. It is abundant in brain tissue, hyaluronic cartilage, and synovial fluid [57]. Its exceptional physicochemical properties have made hyaluronic acid a preferred choice for biomedical applications. However, its rapid enzymatic degradation and poor mechanical strength restrict its wide application [58]. Fortunately, hyaluronic acid possesses an abundance of functional groups such as hydroxyl, carboxyl, and acetyl groups, which allow for various modifications. This enables the enhancement of its properties and the expansion of its application scope [59,60]. For example, Duan’s team successfully developed a dynamic hydrogel using borate bonds. Its injectability and ROS scavenging properties are extremely important in preventing uterine adhesions. In addition, its adjustable degradation rate provides a new idea for the development of anti-adhesion hydrogels [61]. In addition, the aldehydeylation of hyaluronic acid is also a prevalent modification strategy. Yang et al. created a hyaluronic acid-based hydrogel with a dual dynamic network following the aldehyde modification of hyaluronic acid. In this construct, the primary dynamic network is established through the Schiff base reaction between the aldehyde group of aldehyde-modified hyaluronic acid (AHA) and the hydrazide group of 3,3′-dithiobis (propionyl hydrazide) (DTP). The secondary reversible network is formed via ionic interactions between K^+^ and κ-carrageenan (KC). In addition, hydrogen bonding could occur between AHA and KC due to the abundance of hydroxyl groups on their molecular chains. This dual dynamic network provides optimal moist conditions for wound healing, surpassing the benefits of a single dynamic network [62].

### 2.5. Other Polysaccharide-Based Hydrogels

In the research and application field of polysaccharide hydrogel materials, in addition to common polysaccharides like chitosan, cellulose, and gelatin, many other kinds of polysaccharides have been explored and developed. Their unique structures and properties endow hydrogel materials with diverse functions and applications. For example, dextran, as a widely available water-soluble polysaccharide, can be obtained from cereals, seaweed, yeast, and fungal cell walls [63,64]. Compared with other polysaccharides, dextran has excellent uniformity and long-cycling properties similar to polyethylene glycol, which helps to avoid adsorption of serum proteins and clearance of the endothelial reticular system [65], thus showing important application potential in the biomedical field. On this basis, to improve the therapeutic effect of infected wounds, Liang et al. proposed an aerogel-hydrogel duplex hydrogel method and developed an AHB gel consisting of quaternary ammonium polyvinyl alcohol and dextran. This system effectively reduces local inflammation and bacterial infection at the wound site through the release of hydrogen sulfide and the photodynamic antibacterial action of sodium chlorophyll during wound treatment [66]. Furthermore, starch, a natural polysaccharide, is an ideal candidate for biomaterials and packaging materials because of its abundance, sustainability, and low [67]. However, it has the limitations of structural differences, insolubility in cold water, and decomposition during reheating or treatment under acidic conditions. Therefore, various physical or chemical modifications are usually employed to improve the characteristics of starch to realize diverse application needs [68]. Similar to the green starch-based hydrogel prepared by Xu et al., they proposed a Cu-gallic acid-carvacrol nanosphere (CGC NP) functionalization scheme for improved functionality of starch-based hydrogels through coordination interactions, electrostatic interactions, and intramolecular and intermolecular hydrogen bonds. This approach endowed the hydrogel with antioxidant and anti-infection capabilities. At the same time, the hydrogel can completely cover irregular wound surfaces and accelerate wound healing. This is also a new idea for postoperative anti-adhesion [69].

## 3. Structure and Preparation Methods of Polysaccharide-Based Hydrogels

### 3.1. Homogeneous Polysaccharide-Based Hydrogels

**Figure 3:**
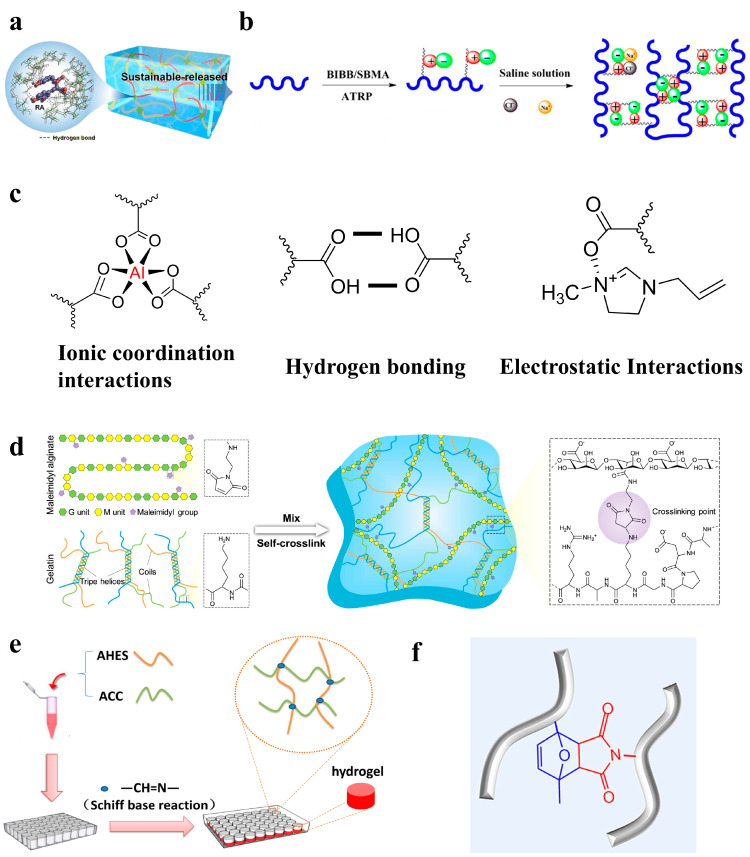
(**a**–**c**) Physical crosslinked hydrogels [71,72,73]: (**a**) hydrogen bonding [71]; (**b**) ionic interactions [73]; and (**c**) ionic coordination interactions, hydrogen bonding, and electrostatic interactions [72]. (**d**–**f**) Chemical crosslinked hydrogels [74,75,76]: (**d**) Michael reaction [74]; (**e**) Schiff base reaction [75]; and (**f**) Diels–Alder click reaction [76].

Homogeneous polysaccharide-based hydrogels usually exhibit a relatively uniform network structure without anisotropy [70]. And they show great application potential in the fields of biomedicine, environmental protection, and advanced materials. As shown in Figure 3, the preparation of these hydrogels primarily involves physical and chemical crosslinking methods.

#### 3.1.1. Physical Crosslinking

Physical crosslinking usually results from physical interactions, such as hydrogen bonds, ionic interactions, electrostatic interactions, and hydrophobic interactions. The hydrogels prepared using physical crosslinking avoid the use of crosslinking agents and possess favorable biocompatibility and degradability. However, the intermolecular forces are usually weak, so there are problems of poor mechanical strength and stability [77,78].

1. Hydrogen bond

As a significant non-covalent interaction, hydrogen bonding plays a vital role in the physical crosslinking process of hydrogels. This interaction is dynamic and capable of re-forming after disruption. In the network structure of hydrogels, the presence of hydrogen bonds effectively hinders the entry of water molecules, thereby slowing the expansion rate of hydrogels [79]. However, the formation of stable and robust hydrogen bonds during the fabrication of hydrogels is a challenge, as water molecules tend to weaken or even break these hydrogen bonds [80]. Gao et al. designed a hybrid hydrogel consisting of the natural compounds rosmarinic acid (RA), chitosan (CS), and polyvinyl alcohol (PVA). Utilizing non-covalent hydrogen bonding interactions, they ingeniously used RA-CS as the active ingredient. Treatment trials performed on a mouse skin wound infection model ultimately revealed that the hydrogel effectively realized its purpose of providing antimicrobial action and facilitating wound healing, indicating its substantial potential for practical application (as shown in Figure 3a) [71]. Moreover, hydrogels formed by hydrogen bonding exhibit excellent self-healing ability. Zhao et al. developed a semi-interpenetrating polymer network hydrogel based on sodium alginate (SA) and polyacrylamide (PAM), with improved lamellar structure and properties. Due to the abundant hydrogen bond network in the hydrogel, the damaged site was able to self-repair under mild conditions, and the original mechanical properties were restored effectively [81].

2. Ionic interaction

Ionic interactions are dynamic interactions between oppositely charged groups. These interactions are primarily established through metal–ligand bonds or the polymerization of oppositely charged monomers. Hydrogels prepared based on ionic interactions are usually characterized by fast response and self-healing. Nevertheless, the creation of tough and self-healing hydrogels via ionic interactions in a straightforward process remains challenging [82]. To address this issue, Yuan et al. proposed a simple synthesis method. They prepared a novel physical hydrogel by incorporating a negatively charged monomer (acrylic acid, AAc) into a solution of a positively charged natural polysaccharide (2-hydroxypropyltrimethylammonium chloride chitosan, HACC). This hydrogel has a reversible high-density dynamic ionic interaction structure, endowing the PAAc/HACC hydrogel with excellent fatigue resistance, ionic conductivity, and self-healing properties [83]. Furthermore, Ye et al. developed a starch-based superhydrophilic zwitterionic polysulfobetaine methacrylate (PSBMA)-modified anti-protein and degradable hydrogel platform. The gelation process of this hydrogel occurred under mild conditions, without the need for high temperatures or chemical crosslinkers, relying only on the ionic interaction between the zwitterionic moieties (as shown in Figure 3b). The results showed that this hydrogel had excellent anti-protein and anti-cell adhesion properties, low cytotoxic activity, and good biocompatibility and biodegradability. Additionally, the cytokine secretion assays substantiated that the hydrogels possess a reduced likelihood of initiating macrophage activation, making them well suited for in vivo biomedical utilization [73].

3. Electrostatic interaction

As a type of weak force commonly found in nature, electrostatic interaction has shown unique advantages in its application in materials science. Compared with ionic hydrogels based on neutral polymer networks without electrostatic interactions, electrostatic polymer network-based ionic hydrogels perform better in terms of light transmittance, ionic conductivity, and mechanical properties [84]. Recently, as shown in Figure 3c, Yan et al. achieved light-curing 3D printing of conductive hydrogels through the utilization of hydrogen bonding, ionic coordination interactions, and electrostatic interactions [72]. In this study, acrylamide (AM) and hydroxyethyl acrylate (HEA) served as crucial photopolymerized active compounds. In addition, the modification of carboxymethyl cellulose (CMC) with methacrylic anhydride (MA) not only enhanced the mechanical properties of the hydrogel but also formed a double network structure. This structure was established through ionic coordination bonds between Al^3+^ and carboxymethyl cellulose, as well as hydrogen bonds within and between the polymer network. This structure endowed the hydrogel with excellent mechanical properties and adhesion. Concurrently, the electrostatic interaction between imidazole chloride salt (1-allyl-3-methylimidazole chloride salt, AMIMCl) and the polymer network facilitated ion transport along the polymer chain, significantly improving the conductivity of hydrogels. Due to its high transparency, strength, tensile strain, thermal stability, and strain sensing properties, the hydrogel holds significant promise in the domain of flexible wearable sensors. This breakthrough paves a novel path for the molding and application of hydrogel materials.

4. Hydrophobic interaction

The hydrophobic interaction is a strong and stable physical interaction. It prompts hydrophobic segments to aggregate into micelle-like structures through intermolecular entanglements, which serve as dynamic physical crosslinking points in the architecture of the hydrogel. When the hydrogel is subjected to external forces and deforms, the originally curled hydrophobic segments can slide against each other and untangle the entanglement. This process is accompanied by energy dissipation, which increases the viscous modulus of the hydrogel [85]. Furthermore, hydrophobic interactions can prevent hydrogel rupture and impart self-healing properties. Therefore, Fredrick et al. prepared a non-toxic and hemocompatible material by conjugating dioctylamine to carboxymethyl cellulose (CMC). The results showed that this hydrogel had moderate rheological properties and that its hydrogel properties can be completely and quickly restored after the strain is released [86].

The mechanical strength of hydrogels can be adjusted by regulating the relative amount of hydrophobic and hydrophilic components, as well as the type and size of the hydrophobic agent. Therefore, it can better improve the properties of hydrogels such as drug delivery, hemostasis, and adhesion [87]. For example, Meng et al. used hydrophobic interactions as possible sacrificial bonds to integrate them into the ionic network of alginate, resulting in silk fibroin-based hydrophobic association (HA) hydrogels with superior performance [88]. This design not only enhanced the mechanical ductility, strength, and toughness of the hydrogel, but also conferred self-healing abilities at room temperature without the need for external stimuli. Both mechanical and rheological measurements showed that the hydrophobic interaction acts as a sacrificial bond in these hydrogels, preferentially breaking before the alginate ion network under the action of external forces. This mechanism dissipates a large amount of energy, thereby improving mechanical properties. Additionally, the presence of hydrophobic interactions enables HA hydrogels to quickly restore their structural integrity after injection. In conclusion, their superior mechanical properties give them great potential for use in the prevention of tendon adhesions.

#### 3.1.2. Chemical Crosslinking

Chemical crosslinking constructs a three-dimensional network through irreversible covalent bonding. The hydrogels prepared based on chemical crosslinking typically exhibit structural stability, robust mechanical properties, and enhanced durability. Nonetheless, they may lead to residual monomers, which can diminish biocompatibility.

5. Michael reaction

The Michael addition reaction is the addition of nucleophiles, such as an allyl anion (known as a Michael donor), to α, β-unsaturated compounds (the Michael receptors) [89]. This reaction is highly selective under physiological conditions and avoids the use of toxic reagents and by-products. Due to these advantages, the Michael addition reaction has been widely used in the biomedical field, especially in the preparation of injectable hydrogels [90]. For example, Yin’s team developed a multifunctional hyaluronic acid-based wound dressing through a Michael addition reaction. Their results show that the dressing not only modulates macrophage polarization, activates angiogenesis, and ultimately promotes wound healing in diabetic mice, but also has antioxidant, antibacterial, and anti-inflammatory effects. It will potentially be used in a wider range of clinical disease models in the future [91]. In addition, Chen et al. developed an innovative injectable hydrogel composed of maleimide alginate and virgin gelatin [74]. As shown in Figure 3d, this hydrogel is unique in that its precursor solution can self-crosslink through a gentle Michael addition reaction, a process that does not rely on catalysts or external energy inputs. The hydrogel is tough and bioadhesive, maintaining high integrity and adhesion to pig skin under severe mechanical stresses, such as bending, twisting, and exposure to warm and boiled water. Moreover, the hydrogel can mimic the extracellular matrix, provide a suitable growth environment for cells, and promote cell proliferation and remodeling. To verify its practical application potential, the team conducted an experiment on diabetic skin wound healing. And the results showed that the hydrogel could significantly promote the wound healing process. Therefore, this hydrogel has great application prospects in the healthcare field, providing new biomaterial options for tissue engineering and wound management.

6. Schiff base reaction

Schiff base reaction, also known as imine formation reaction. It refers to a type of chemical reaction between primary amines and carbonyl compounds, such as aldehydes or ketones, catalyzed by acids or bases to form imines. During this process, intermediate imine ions are formed and then react with nucleophiles to form target imine products [92]. The reaction proceeds rapidly under mild conditions, without the need for metal catalysts. Due to its reversibility, pH responsiveness, high reactivity, and biocompatibility, the Schiff base reaction offers distinctive advantages in the biomaterials domain. In particular, hydrogels prepared based on the Schiff base reaction not only have inherent self-healing ability but also exhibit intelligent characteristics like injectability. Therefore, these hydrogels have shown significant potential in various fields [93]. For example, Liu et al. synthesized aldehyde hydroxyethyl starch (AHES) by oxidizing hydroxyethyl starch [75]. Subsequently, they prepared aminocarboxymethyl chitosan (ACC) by grafting ethylene diamine onto carboxymethyl chitosan and increasing the number of amino groups. As shown in Figure 3e, using the Schiff base reaction between the aldehydes and amino groups, they further formed two-component AHES/ACC hydrogels. Experimental results showed that the hydrogel supported cell activity and proliferation in vitro and exhibited excellent hemostatic effect and good biocompatibility in vivo. Consequently, AHES/ACC hydrogels have the potential to be ideal tissue adhesives and hemostatic wound dressings for future clinical applications. Furthermore, Lee et al. developed a chitosan-based hydrogel using the Schiff base reaction between the polyaldehyde group (NIPAM-co-FBEMA) and amine groups on chitosan [94]. Thanks to the dynamic covalent bonds, this SC/PNF hydrogel exhibited a range of intelligent properties, including pH responsiveness, reversible sol–gel transition, injectability, and self-healing ability. This hydrogel not only degraded rapidly but also had a long-term bactericidal effect, thus showing great potential for application in the biomedical field.

7. Diels–Alder (DA) reaction

The Diels–Alder (DA) reaction is a highly selective [4+2] cycloaddition reaction involving a chemical reaction between electron-rich and electron-poor diene. Notably, this reaction can be significantly accelerated in the water phase due to the enhanced hydrophobic effect [95]. In addition, the Diels–Alder reaction is widely applied in the field of the production of biomaterials combined with bioactive molecules. These materials can be used to construct hydrogel networks from natural or synthetic biomaterials, enabling precise control of hydrogel networks. The Diels–Alder reaction occurs under gentle reaction conditions with high yields and no by-products or reagent contamination in the process. These features make the Diels–Alder reaction an invaluable tool in the synthesis of hydrogel materials [96]. For example, Hu et al. prepared an injectable hyaluronic acid hydrogel using the Diels–Alder reaction (as shown in Figure 3f). It was loaded with human umbilical cord mesenchymal stem cells (UCMSCs) and aimed at promoting endometrial regeneration and restoring fertility [76]. They employed a minimally invasive technique to deliver a precursor solution directly into the uterine cavity. The low viscosity and high fluidity of the precursor solution during the injection effectively minimized stem cell damage from shear forces, thereby maintaining high cell viability. Simultaneously, the solution can fully permeate the tissues and adapt to the complex morphology of the uterine cavity. This in situ hydrogel not only acted as a mechanical barrier to isolate damaged areas but also served as a reservoir for the continuous release of therapeutic cytokines and bioactive mediators. In vitro experiments demonstrated the beneficial effects of injectable hydrogels containing UCMSCs on cell proliferation, migration, angiogenesis, and antifibrosis. In vivo experiments in a rat endometrial injury model showed that the hydrogel could promote endometrial regeneration, collagen remodeling, enhanced endometrial receptivity, and the restoration of fertility. This system has shown potential in regenerating damaged endometrium and restoring fertility. Therefore, it is considered a promising strategy for treating endometrial injuries and fertility decline caused by intrauterine adhesions.

### 3.2. Janus Hydrogel

**Figure 4:**
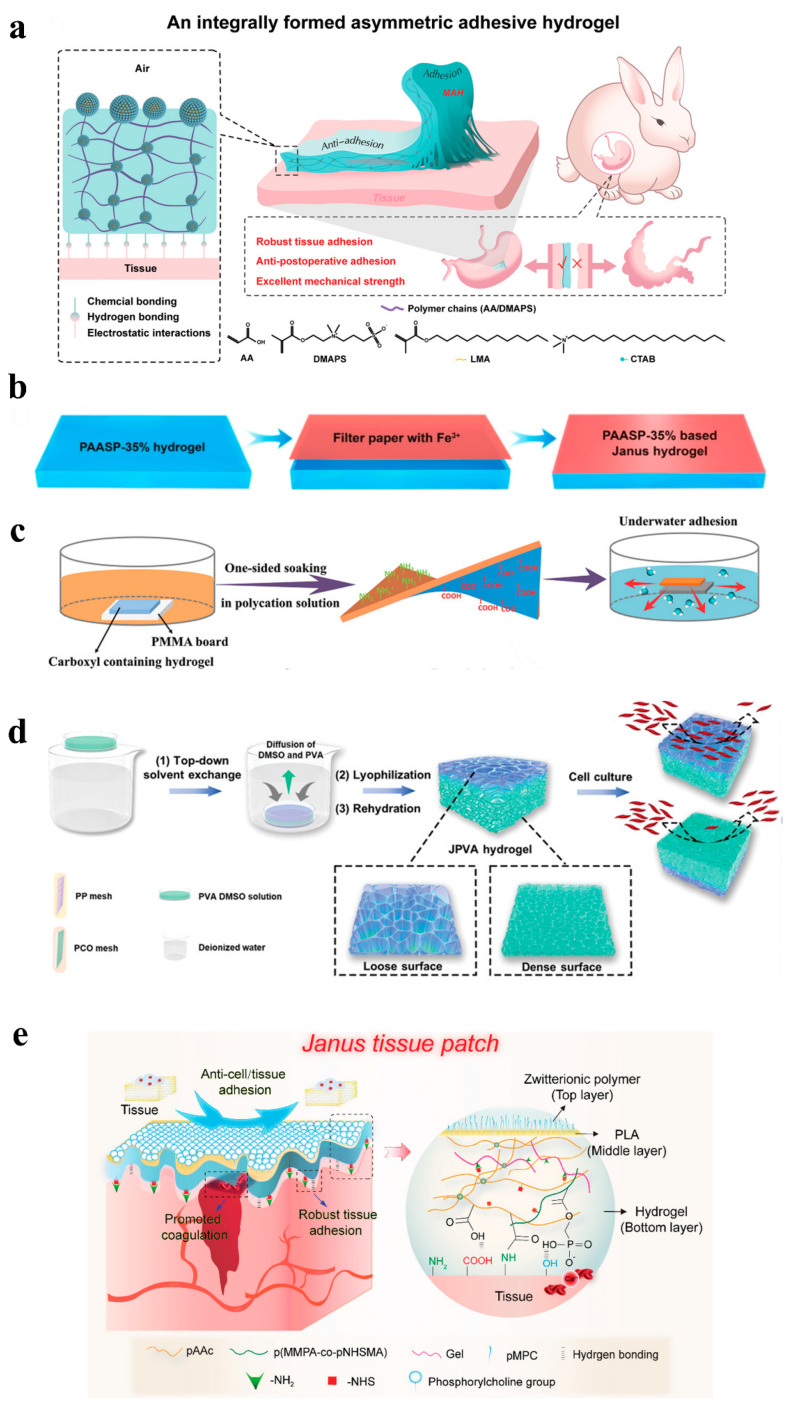
(**a**) One-step method [101]; (**b**–**d**) unilateral processing [102,103,104]; (**e**) layer-by-layer method [105].

Most previously reported hydrogel bioadhesives have been designed with a focus on either solely preventing postoperative adhesion [97,98] or enhancing tissue adhesion strength [99,100]. These hydrogels often result in non-discriminatory bilateral adhesions, which can lead to significant adhesion between normal and damaged tissues after surgery. To address this, there is a need to ingeniously combine materials with varying properties to create Janus hydrogels that strongly adhere to damaged tissues while preventing adhesion to normal tissues, thereby effectively realizing a postoperative anti-adhesion effect. At present, several methods for the preparation of Janus hydrogels have been reported (Figure 4): (1) selecting appropriate materials to prepare using a one-step method of integral formation [101]; (2) unilateral processing of the prepared hydrogel, such as unilateral ion sealing and solvent immersion [102,103,104]; and (3) forming different physical and chemical properties between layers by multilayer composite processing [105].

#### 3.2.1. Unilateral Processing

The unilateral treatment method is a common technique for the preparation of Janus hydrogels, imparting distinct properties to one side of the hydrogel through selective treatment (Figure 4b–d) [103,104]. This approach is simple to operate and easy to control and enables the realization of multiple functions, including anti-adhesion, drug delivery, and sensing. For example, Jia et al. achieved single-sided adhesion by spraying a cationic solution onto one side of the powder. It uses the interaction between the cation and the negatively charged groups on the surface of the material to eliminate viscosity [106]. This method was not only easy to operate but also highly controllable. By selecting different cationic solutions or adjusting the concentration of the spray, the strength of the adhesion can be precisely controlled to suit different application needs. Its unique Janus adhesion characteristics provided an effective solution for postoperative adhesion while avoiding adhesion to the surrounding tissue. In addition, fast-Janus-gelation (FJG) powder has robust mechanical properties to adapt to the movement of different organs and degrade safely in vivo. Its convenient storage and delivery further enhanced its potential for application in minimally invasive surgery. In addition, Cui et al. successfully prepared carboxyl-containing hydrogels with excellent performance using an innovative gradient polyelectrolyte complexation strategy (as shown in Figure 4b). It solved the problem of carboxyl group instability in a normal saline environment [103]. They immersed the top surface of a carboxyl-based polymer hydrogel in a polycationic solution, while the bottom surface was designed to avoid contact with the solution, thus creating a physical barrier. This hydrogel replaces traditional suturing methods, with the sticky side firmly adhering to the damaged area, while the other side effectively prevents adhesion. In vivo experiments have confirmed that this hydrogel can degrade and repair the perforated stomach well.

#### 3.2.2. Layer-By-Layer Method

The layer-by-layer assembly method is a strategy for constructing Janus hydrogels by alternating the deposition of different layers of material (as shown in Figure 4e) [105]. Although this method is complex and expensive, it can create Janus hydrogels with multifunctionality, which are suitable for a wide range of applications. Take the study of Yang’s team, who took inspiration from the gradient structure of natural skin to develop an innovative asymmetrical double-layer dressing [107]. The inner layer of this dressing is a striated spore polysaccharide (BSP) sponge, designed for immediate hemostasis. The outer layer is a dense carboxymethyl chitosan (CMCS) hydrogel loaded with panax ginseng saponin (PNS) to promote angiogenesis during tissue reconstruction. In vivo results showed that the BSP-PNS@CMCS dressing not only significantly reduced the massive bleeding, but also effectively inhibited the inflammatory response and accelerated angiogenesis and collagen deposition in infectious full-thickness skin wounds, thereby promoting rapid wound healing. This hydrogel dressing combines the dual functions of hemostasis and angiogenesis. It provides a new solution for the treatment of hemorrhagic wounds, demonstrating the great potential of biomedical engineering in wound management. Furthermore, a novel Janus hydrogel dressing was designed for treating oral ulcers [108]. This dressing features a bilayer dual-network structure with a tough layer and an adhesive layer. The tough layer consists of polyethylene glycol diacrylate (PEGDA) and polyvinyl alcohol (PVA) to provide mechanical strength and flexibility. The adhesive layer consists of N-[triple-(hydroxymethyl) methyl] acrylamide (THMA) and chitosan (CS) to provide excellent wet adhesion properties. The adhesive surface of this Janus-structured hydrogel adheres firmly to oral mucosal wounds, withstanding the pressure of oral movements and preventing tissue adhesion while avoiding irritation from external factors. Therefore, this hydrogel is expected to provide a new idea for the prevention of postoperative adhesions.

#### 3.2.3. One-Step Method

Traditional multi-step hydrogel preparation methods often lead to poor repetition and controllability of product performance. Additionally, the complexity of these processes and the challenges associated with interlayer adhesion constrain the practicality of hydrogel production. To overcome these challenges, researchers began to explore a one-step assembly method to prepare asymmetric hydrogels (as shown in Figure 4a) [101]. For example, He et al. proposed a simple and universal strategy for the preparation of bilayer hydrogels in one step by increasing the viscosity difference between the two layers of precursor solutions [109]. Specifically, they precisely modulated the viscosity contrast by varying the concentration of a thickening agent (usually a macromolecular substance) in one of the precursors. This method ensured the stability of the bilayer structure during formation and fixed the structure by polymerization. In addition, they performed a local monomer exchange in the interface region of the bilayer structure, which enhanced the interlayer bonding strength. This simple and versatile approach provides a new way of designing Janus hydrogels and broadens their applications.

## 4. Application in Preventing Postoperative Adhesion

### 4.1. Peritoneal Adhesion

Peritoneal adhesion is a common long-term complication after abdominal surgery and was first described by Richard Bright in 1835 [110]. It commonly forms between the surfaces of organs and the abdominal wall [111]. It arises from detrimental processes, such as coagulation, inflammation, and fibrinolysis [112]. It can result in serious symptoms, such as adhesive small bowel obstruction and chronic pain [113]. Such symptoms significantly impact the health and economic development of individuals and society.

**Figure 5:**
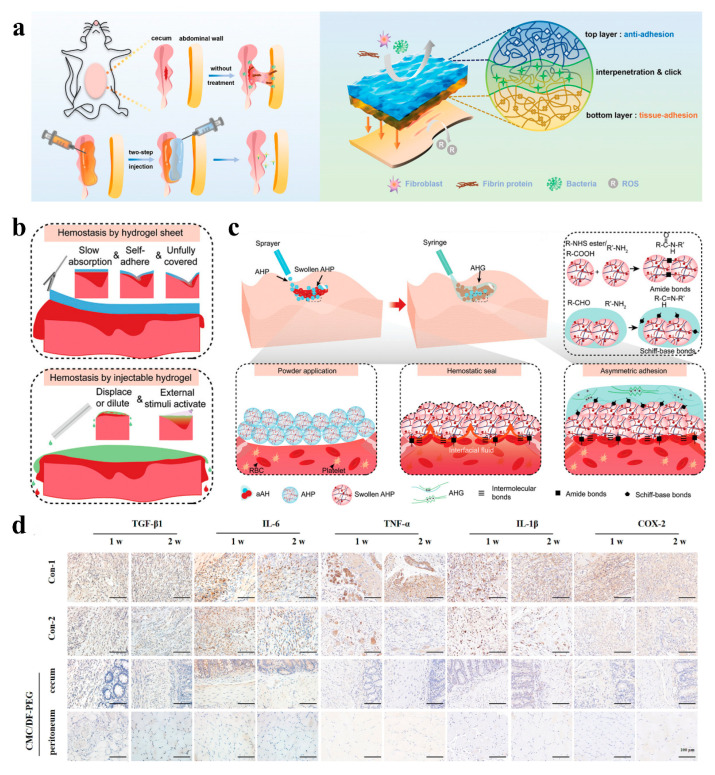
(**a**) Schematic representation of the AAB-hydrogel structure [119]; (**b**) graphical representation of hydrogel tablets and injection hydrogels in laparoscopic surgery [120]; (**c**) schematic overview of AHP and AHG crosslinking to form an asymmetric adhesion hydrogel and adhere to tissues [120]; (**d**) representative immunohistochemical staining of different groups at 1 and 2 weeks after surgery [115].

With the development of technology, non-Janus structured polysaccharide-based hydrogels have shown promising potential for the application of peritoneal adhesion recently. Polysaccharide materials like chitosan are instrumental in postoperative adhesion prevention. Chitosan can reduce the adhesion of fibroblasts, but its poor solubility limits further clinical application. To address this limitation, carboxymethyl chitosan, as a derivative of chitosan, is usually used to prevent postoperative adhesion [114]. For example, Wang et al. prepared an injectable hydrogel through the Schiff base reaction between carboxymethyl chitosan (CMC) and dialdehyde functional polyethylene glycol (DF-PEG). This hydrogel can form in situ without external stimulation and crosslinking agents, thereby preventing postoperative adhesion by balancing the expression of t-PA and PAI-1, regulating the dynamic balance of the ECM, and reducing the inflammatory response (Figure 5d) [115].

In addition, the Janus polysaccharide-based hydrogel also shows unique advantages in the prevention of peritoneal adhesions (Figure 5). Among these, the zwitterionic material is composed of an equal number of positive and negative charges at the molecular level and exhibits excellent antifouling properties due to its superhydrophilicity. Besides, the zwitterionic material can also effectively prevent infection and inflammation by resisting bacterial adhesion [116,117]. Based on the multiple advantages of polysaccharides, the combination of zwitterionic materials with polysaccharide materials has great potential in the biomedical field [118]. Zhao et al. prepared a bottom hydrogel based on gallic acid-modified chitosan (GACS) and aldehyde-modified dextran (Dex-CHO) using the Schiff base reaction. And then, they formed an aldehyde-modified zwitterionic dextran/carboxymethyl chitosan (Dex-SB-CHO/CMCS)-based hydrogel on the surface to construct an asymmetric hydrogel with an antifouling top layer (Figure 5a). The anti-adhesion efficacy of this asymmetric hydrogel has been confirmed through animal experiments [119]. In addition to developing preventive materials, it is also necessary to focus on rapid hemostasis to reduce the risk of adhesions. More than 2 million people are killed each year due to uncontrolled bleeding, so rapid hemostasis is essential in postoperative environments. Yang et al. developed an asymmetric hydrogel by tightly combining a viscous hydrogel powder and an anti-adhesive hydrogel through the Schiff base reaction. This powder can immediately and firmly adhere in the appropriate position, absorb the interfacial fluid, and concentrate red blood cells and platelets to accelerate hemostasis. Then, hydrogel was injected over the powder to form an anti-adhesion layer to prevent adhesion. In addition, the prepared hydrogel is also compatible with minimally invasive surgery, expanding the possibilities for surgical applications (Figure 5b,c) [120].

### 4.2. Intrauterine Adhesions

**Figure 6:**
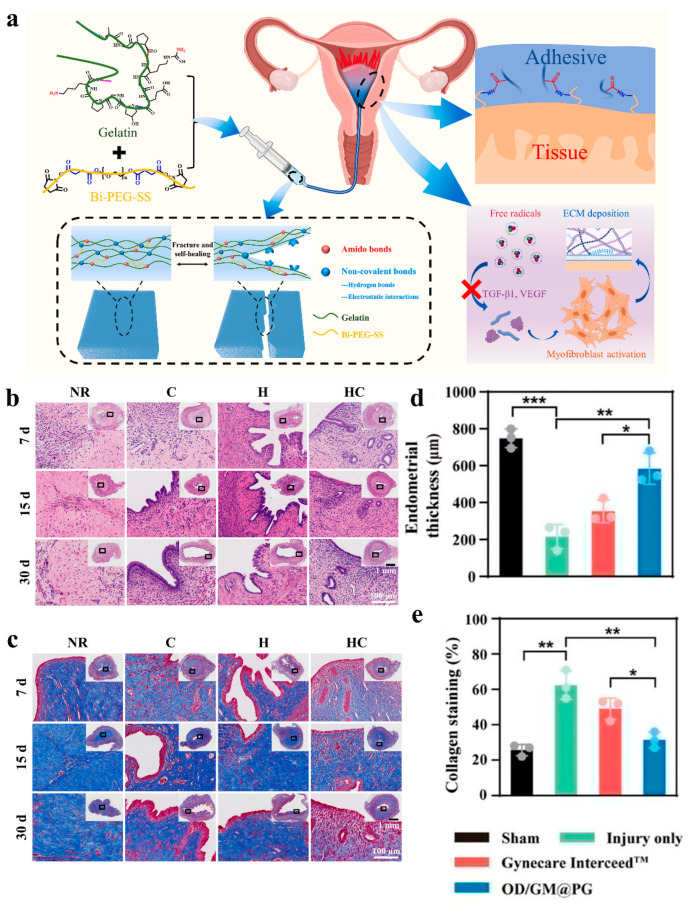
(**a**) Engineering of a self-repairing adhesive hydrogel with antioxidant properties for the prevention of IUA [136]. (**b**,**c**) Representative images of the uterus from different treatments using H&E and Masson trichrome staining at 7, 15, and 30 days after surgery. Among these, UCMSCs suspension (Group C), hydrogel precursor solution (Group H), or hydrogel precursor solution containing UCMSCs (Group HC) were injected into the damaged uterine horn. Rats that received natural repair (NR) without any treatment were used as control groups [76]. (**d**,**e**) Endometrial thickness and collagen staining in different dressing treatment groups. Values in (**d**,**e**) represent the means ± SD [*n* = 3 in (**d**,**e**)]. Statistical significance was calculated via one-way ANOVA with Tukey’s test: * *p* < 0.05; ** *p* < 0.01; *** *p* < 0.001 [138].

Intrauterine adhesion, also known as Asherman syndrome, is a condition that was originally described by Joseph Asherman in 1948 [121]. It is a serious gynecological condition characterized by partial or complete adhesions of the uterine cavity and/or cervical canal. It often leads to menstrual abnormalities, infertility, recurrent miscarriages, and other pregnancy complications [122], which lead to serious negative effects on a woman’s physical and mental health. Studies have indicated that the incidence of intrauterine adhesion is higher following secondary abortions compared to early pregnancies. Therefore, clinicians should avoid terminating pregnancy in the second trimester to minimize the risk of intrauterine adhesion when addressing pregnancy problems [123]. Intrauterine adhesions are usually caused by trauma or infection, leading to a fibrotic process in the endometrium, where endothelial cells transform into mesenchymal cells, thereby promoting the production of fibrotic molecules and ultimately resulting in endometrial fibrosis [124,125]. The core of this process is the proliferation of myofibroblasts and the excessive accumulation of the extracellular matrix, which impairs tissue and organ function [126]. At present, the main treatment methods for intrauterine adhesion include hysteroscopic adhesiolysis [127], intrauterine devices, Foley balloon catheters [128], postoperative hormone therapy, and anti-adhesive agents [129], but these treatments have limited efficacy. Recently, cell therapy has received much attention as an emerging therapeutic method [130]. A variety of scaffolds and hydrogels have been developed to extend the retention time of stem cells and their derivatives [131,132]. However, for clinical applications, hydrogels are advantageous because they can be introduced into the body in a minimally invasive manner, thereby reducing surgical time and the risk of infection (Figure 6) [76].

Because the shape and size of the uterine cavity vary from person to person, the hydrogel used to prevent intrauterine adhesion needs to self-adjust to the different uterine cavity [133]. The self-repairing hydrogel can be injected into the uterine cavity via a needle and spontaneously restore its volume strength without any external stimulation [134,135]. Therefore, as shown in Figure 6a, Feng et al. prepared a series of self-healing hydrogels with adhesion properties using bis-ethylene glycol N-hydroxysuccinimide active ester (Bi-PEG-SS) and gelatin. And they confirmed their efficacy in preventing intrauterine adhesion through in vivo experiments [136]. Although stem cell therapy has been widely used in clinical treatment, there are still challenges such as low delivery efficiency and easy impairment of stem cell activity in the delivery process. To address this, Hu et al. prepared an injectable hyaluronic acid hydrogel using the Diels–Alder reaction, and human umbilical cord mesenchymal stem cells were loaded [76]. This hydrogel could be introduced into the uterine cavity through a minimally invasive method and was able to adapt to complex shapes. Experiments showed that the hydrogel exhibited an antifibrotic effect and promoted endometrial regeneration, which is expected to become a potential therapeutic strategy for patients with intrauterine adhesions (Figure 6b,c).

Additionally, electrospinning technology has received extensive attention in the biomedical field due to its excellent mechanical properties and structure similar to that of the extracellular matrix [137]. This technique is often combined with hydrogels to enhance their properties. Based on these advantages, Yi et al. innovatively combined a hydrogel with an electrostatic spun film to create a new biomimetic microstructured bioadhesive. The hydrogel layer consists of catechol-modified oxidized hyaluronic acid (OD) and methacrylic acid gelatin (GM), while the electrostatically spun layer is a mixture of poly(ε-caprolactone) (PCL) and gelatin. According to the experimental results, this new adhesive can effectively reduce the degree of endometrial fibrosis, prevent intrauterine adhesions, and be used to treat endometrial injuries (Figure 6d,e) [138]. This research demonstrated the great potential of electrospinning technology for hydrogel modification and biomedical applications.

### 4.3. Tendon Adhesions

**Figure 7:**
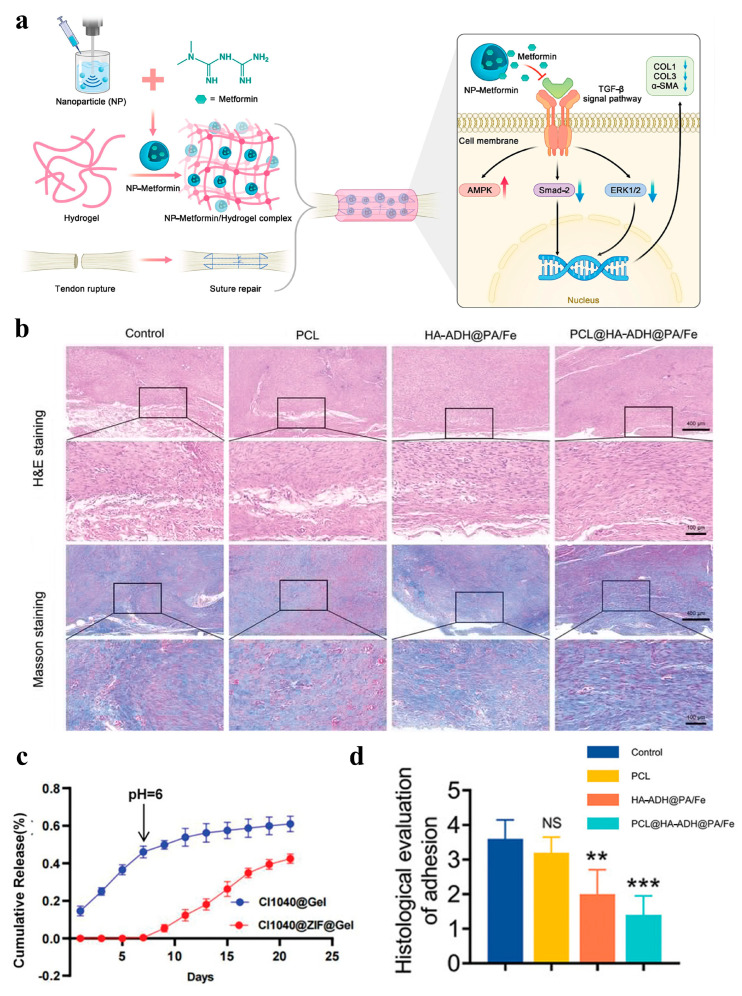
(**a**) Schematic diagram of metformin sustained-release system generation and metformin reduced adhesion mechanism: Validation of metformin in reducing tendon injury-induced adhesions by constructing a slow-release system of metformin to cover the wound site using nanoparticles/hydrogels; Metformin application inhibited the Smad and MAPK pathways of TGF-β1 and decreased the protein expression levels of Col1, Col3 and α-SMA, while activating the AMPK pathway [148]; (**b**) H&E and Masson staining of the tendon tissue [149]; (**c**) the release curves of CI1040 @ Gel and CI1040@ZIF@Gel [146]; (**d**) histological evaluation of peritendinous adhesions based on H&E staining images. Data are computed as average ± SD, and the between-group variation was determined by one-way ANOVA with Tukey posthoc test (*n* = 5; “NS” represents nonsignificance; “**”, and “***” represents statistical significance when setting the alpha level to 0.01, and 0.001, respectively) [149].

Tendon adhesion is one of the most common postoperative complications after tendon injury [139], potentially impeding normal tendon function, diminishing flexibility, and compromising overall motor performance. Tendon healing consists of three overlapping phases, namely acute inflammation, proliferation, and remodeling, which are time-dependent and co-regulated by different cells and cytokines. During the acute inflammatory phase, inflammatory cells rapidly invade the damaged site. Then, in the proliferative phase, fibroblasts proliferate and synthesize the extracellular matrix, resulting in excessive collagen deposition. Finally, the remodeling phase begins, characterized by the reorganization of new collagen and the maturation of fibrous tissue [140,141,142,143]. The occurrence of tendon adhesion is closely related to the imbalance between endogenous and exogenous healing processes. Its specific mechanism involves inflammation, oxidative stress, and excessive fibrosis. Among these, external healing mainly occurs in the early stage of healing, with fibroblasts and inflammatory cells migrating to the damaged site and proliferating to form adhesions, while internal healing involves the proliferation of internal tendon cells, the production and reorganization of the extracellular matrix, and the promotion of new blood vessels [143,144]. When this balance is broken, it leads to the occurrence of tendon adhesions. Traditional treatments mainly include drugs and revision surgery, but both have certain limitations and are not ideal [145]. With the continuous progress of tissue engineering technology, it is important to inhibit the formation of adhesions by introducing physical barriers, such as electrospinning nanofibers or hydrogels (Figure 7) [98].

Researchers have effectively inhibited fibroblast proliferation by incorporating anti-adhesion drugs into hydrogels. For example, Wu et al. chose CI1040 as an anti-adhesion drug, loaded it into ZIF-8, and incorporated it into oxidized hyaluronic acid/N-carboxyethyl chitosan (OHA/CEC) hydrogel. This hydrogel not only acted as a physical barrier to prevent the formation of adhesions, but also further inhibited the proliferation of fibroblasts through the sustained release of the drug (as shown in Figure 7c) [146]. TGF-β1 is an important factor in tendon adhesion. Previous studies have shown that metformin can effectively inhibit TGF-β1-induced fibrosis [147]. Because the hydrogel–nanoparticle system can locally and slowly release drugs, Li et al. loaded metformin on nanoparticles and encapsulated complexes into the hydrogel to develop a hydrogel–nanoparticle system. This system can effectively reduce tendon adhesion by regulating the Smad and MAPK-TGF-β1 signaling pathways (Figure 7a) [148].

Ideal biological materials for preventing tendon adhesions should have multiple functions such as biocompatibility, biodegradability, as well as self-healing, anti-inflammatory, and antibacterial properties. However, there is still a lack of biomaterials with comprehensive functions for the prevention of tendon adhesions. Therefore, developing a biomaterial with multifunctional properties is of great significance for the prevention of tendon adhesions. Yao et al. prepared a bilayer Janus patch with multifunctional HA-ADH@PA/Fe hydrogel as the inner layer and electrospinning PCL as the outer layer. This patch not only effectively inhibited the development of tendon adhesions, but also promoted the polarization of macrophages to the anti-inflammatory phenotype. It showed good anti-inflammatory effects and provided an innovative material choice for the treatment of tendon adhesions (Figure 7b,d) [149].

### 4.4. Pericardial Adhesions

**Figure 8:**
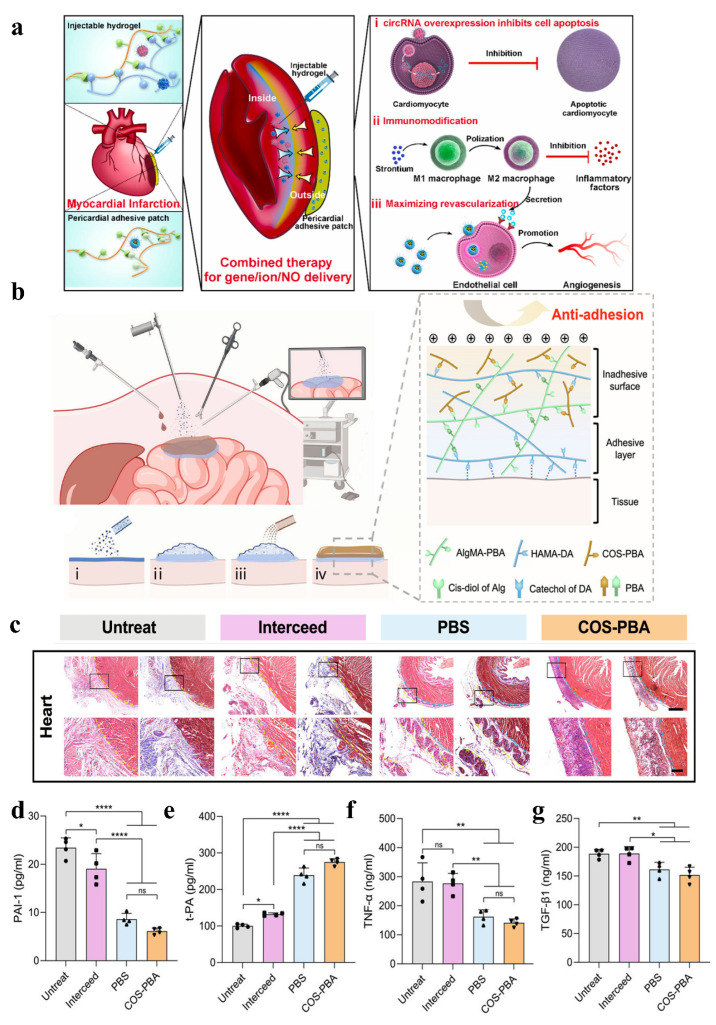
(**a**) Schematic illustration of the integrated therapy approach: combined administration of lipo/pcircRNA and SiSr-loaded injectable HL hydrogel for immune activity, alongside a lipo/NO nanodrug-loaded wet adhesive hydrogel cardiac patch for intramyocardial gene/ion delivery and epicardial NO delivery to restore cardiac function after myocardial infarction [154]. (**b**) Application of FJG powder in endoscopic surgery, gelation process and network diagram: (i) FJG powder on the tissue surface through the delivery device; (ii) FJG powder hydrates with surface moisture to form an adhesive hydrogel layer; (iii) spray COS-PBA solution on the upper surface of the unhydrated powder; (iv) formation of the Janus hydrogel barrier. (**c**) Representative H&E staining (left) and Masson trichrome staining (right) of day 14 tissue sections. The yellow dotted line indicates the boundary between the proliferative adhesion and the organ or the implanted material, and the blue dotted line indicates the boundary between the implanted material and the organ. (**d**–**g**) The serum concentrations of PAI-1, t-PA, TNF-α, and TGF-β1 detected by ELISA. Data are shown as mean ± SD and compared using one-way ANOVA followed by Bonferroni’s post hoc test. *, **, and **** indicate *p* < 0.05, *p* < 0.01, and *p* < 0.0001, respectively. ns indicates no significant difference [106].

During cardiac surgery, damage to the epicardium and pericardium, as well as surgical stimulation, often leads to the formation of pericardial adhesions. These adhesions not only prolong the operation time and increase surgical risks, but may also affect the normal function of the heart and increase the incidence of postoperative complications [150,151]. The formation of pericardial adhesions involves several factors, including the loss of pericardial mesothelial cells, adhesion of fibrin in the absence of these cells, a reduction in normal fibrinolytic activity, and local inflammation, all of which contribute to the occurrence of pericardial adhesions [152]. To prevent pericardial adhesions, various clinical strategies are employed, including the application of a rapid hydrogel barrier on the epicardium to prevent tissue adhesion to other thoracic organs (Figure 8a) [153,154].

As a natural polysaccharide, chitosan has been widely used in the study of preventing postoperative adhesions [28]. Daroz et al. studied the effect of heat-sterilized carboxymethyl chitosan hydrogel on the prevention of pericardial adhesions after sternotomy. Animal experiments and histological morphological analysis have shown that the hydrogel could reduce the severity of pericardial adhesions and the occurrence of complications after surgery [155]. Subsequently, Lopes et al. dissolved keratinocyte growth factor into a heat-sterilized carboxymethyl chitosan hydrogel. And the combination of the two showed an excellent synergistic effect, which could further enhance the prevention of pericardial adhesions [156].

Traditional hydrogels mainly act as physical barriers. Although they can prevent bleeding and pericardial effusion, they cannot reduce oxidative stress and inflammatory responses. Therefore, it is necessary to design hydrogels that can prevent postoperative pericardial adhesions and reduce inflammation under oxidative stress conditions [157]. Previous studies have proved that exosomes from induced pluripotent stem cell-derived cardiomyocytes (iCMs) can improve cardiac function [158], but their heart-specific distribution is limited due to rapid uptake by macrophages and subsequent accumulation in the liver and spleen [159]. To solve this problem, Wang et al. packaged cardiomyocyte exosomes derived from induced pluripotent stem cells (iCMs-EXO) in hyaluronic acid-g-(2-aminoethylmethacrylate hydrochloride-dopamine) (HAD) hydrogel of asymmetric adhesion. It effectively inhibited postoperative pericardial adhesion by reducing oxidative stress and inflammation [157]. In addition, with the increasing use of endoscopic surgery, the development of barriers that are compatible with this technique is also particularly important. To solve the contradiction among endoscopic delivery, Janus adhesion, and mechanical properties, Jia et al. developed a sprayable powder that can form Janus hydrogel in situ. This hydrogel can achieve anti-adhesion effectiveness in different locomotor organs and has shown great potential in both clinical and commercial applications (Figure 8b–g) [106].

### 4.5. Epidural Adhesions

**Figure 9:**
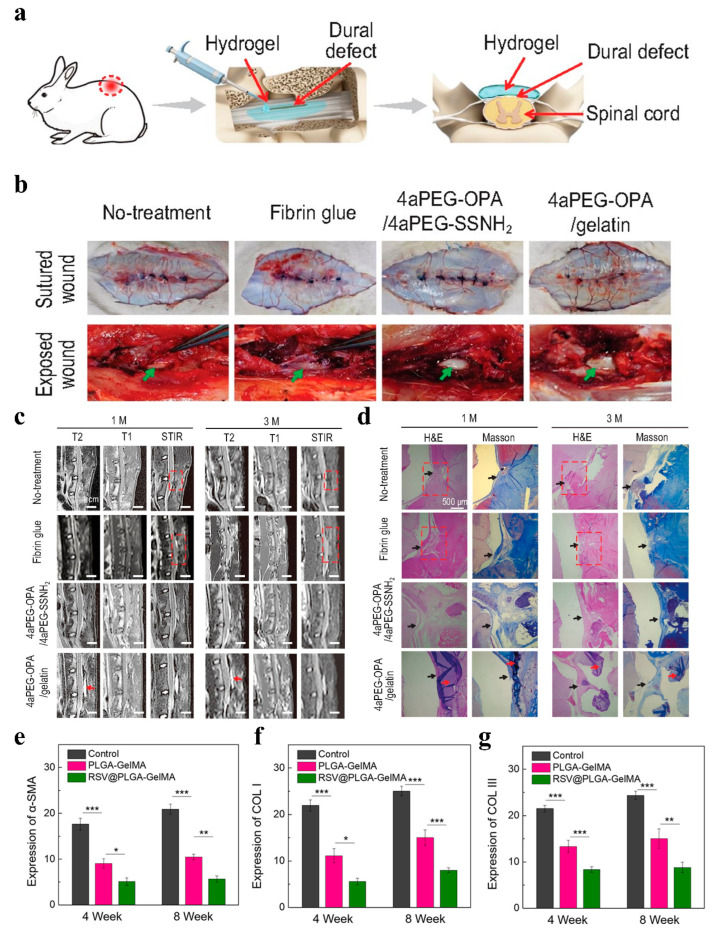
(**a**) Schematic diagram of sealing and preventing undesired postoperative adhesion in the dura mater spinalis of rabbits. (**b**) Visual inspection of surgical sites to evaluate epidural adhesion at 1 month after different treatments. Green arrows represent the dura mater. (**c**) The lumbar MRI results of rabbits in the no-treatment, fibrin glue, 4aPEG-OPA/4aPEG-SSNH2, and 4aPEG-OPA/gelatin groups. (**d**) H&E and Masson’s trichrome staining for different groups. Red arrow: residual hydrogels; red dashed box: area of epidural adhesion [167]. (**e**–**g**) Quantitative analysis results of α-SMA, COL I, and COL III expression. * *p* < 0.05, ** *p* < 0.01, *** *p* < 0.001. (*n* = 3) [164].

Epidural adhesions in the epidural space are mainly caused by local trauma and inflammation, leading to pain and the potential for failed back surgery syndrome, and represent a significant obstacle to the success of spinal surgery [160]. The formation of epidural adhesions is closely related to the proliferation of fibroblasts and the excessive deposition of the extracellular matrix. During laminectomy, many inflammatory cytokines and growth factors are produced at the surgical site. These further stimulate the proliferation of fibroblasts and the deposition of the extracellular matrix, eventually leading to the formation of epidural adhesions [161]. At present, numerous strategies have been developed to prevent epidural adhesions, such as the development of anti-inflammatory drugs, the improvement of surgical techniques, and the addition of barriers between the epidural space and muscles. However, the clinical efficacy of drug treatment and improved surgical methods remains limited. In contrast, polymer materials as a physical barrier can effectively isolate inflammatory substances and be used as drug carriers to realize the slow release of drugs, so as to achieve stable and prolonged treatment effects (Figure 9) [162,163,164].

Epidural fibrosis usually leads to adhesions, and commonly used anti-adhesive and antifibrotic agents often lack sufficient stability. In contrast, 1,4-butanediol diglycidyl ether crosslinked hyaluronic acid (cHA) has better stability, but its antifibrotic effect requires further investigation. Hsu et al. found that cHA can inhibit the migration and proliferation of primary tendon cells, downregulate the expression of fibronectin, and modulate the expression of MMP3, collagen-1, and LC3-II in a dose-dependent manner. These results suggest that cHA may inhibit fibrosis in primary tendon cells by reducing autophagic activity, thereby having the potential to prevent epidural fibrosis and failed dorsal surgery syndrome after spinal surgery. Therefore, cHA may be a promising material for the prevention of epidural fibrosis [165]. In addition, hyaluronic acid is susceptible to tissue-mediated enzyme degradation, which may lead to insufficient stability in the body. To overcome this defect, Lin et al. prepared a composite hydrogel of polygalacturonic acid and hyaluronic acid using the Schiff base crosslinking reaction. This hydrogel showed a good effect on preventing epidural fibrosis in animal experiments. However, it degrades slowly in vivo, and residues might induce inflammatory reactions [166]. Therefore, there is a demand for the development of a new type of epidural anti-adhesion hydrogel with good biocompatibility and a low swelling ratio. Here, as shown in Figure 9a-d, Cheng et al. prepared an injectable and low-swelling hydrogel sealant based on gelatin and phthalatedehyde (OPA), which avoided nerve compression in the limited spinal and intracranial space, effectively reducing epidural fibrosis and postoperative adhesions [167]. In addition, combining hydrogels with microspheres is an effective strategy for preventing epidural fibrosis. In a rat laminectomy model, this hydrogel prevented postoperative epidural fibrosis through the TGF-β/Smad signaling pathway (Figure 9e–g) [164].

Furthermore, Janus hydrogel shows great potential in spinal surgery, offering suitable mechanical strength and elasticity for the postoperative environment of spinal surgery. This is crucial for the stable prevention and treatment of epidural adhesion, a common complication after spinal surgery. The dual characteristics of the Janus hydrogel enable it to effectively isolate the epidural space from the surrounding musculature, thus effectively reducing the accumulation of adhesive material. One advantage of Janus hydrogel over traditional hydrogel is its adaptability to the complex postoperative environment. Although the polysaccharide-based Janus hydrogel is still in the early stages of preventing epidural adhesions, its great application potential will be gradually released with further study of Janus hydrogel properties. With the continuous advancement of related research, Janus hydrogel is expected to become an important auxiliary material in spinal surgery.

## 5. Commercial Products

At present, various anti-adhesion products based on polysaccharide materials have been introduced to the market. Table 2 lists the details of several popular products currently on the market.

Seprafilm^®^ is a hydrogel membrane composed of carboxymethylcellulose and hyaluronic acid, frequently utilized in abdominal and pelvic surgeries to prevent adhesions [168,169,170,171]. It remains effective even in the presence of blood, enhancing its practicality during surgical interventions [172]. When applied in vivo, it transforms a hydrogel within two days, firmly adheres to the damaged site, and gradually degrades within a month [173]. It not only eliminates the need for sutures but also helps to reduce the risk of postoperative infection. However, its rigidity and brittleness in wet environments limit its use, especially in laparoscopic surgeries [174].

Interceed^®^ is an anti-adhesion membrane composed of oxidized regenerated cellulose, used in pelvic surgeries like obstetrics and gynecology. It sticks well to wounds without sutures and is fully absorbed within two weeks. Compared with Seprafilm^®^, Interceed^®^ has better flexibility and adhesion, but its efficacy is reduced in the presence of blood. Therefore, it is necessary to ensure that the blood at the surgical site has been cleared before using Interceed^®^; this undoubtedly increases the complexity and difficulty of the operation [175,176]. Oxiplex/AP^®^ is a transparent, single-use, flexible barrier made of carboxymethyl cellulose and polyethylene oxide, commonly used in intrauterine and abdominal surgeries. Its clarity allows for clear visualization of surgical and neural structures, and it has excellent tissue adhesion, enabling it to adhere firmly to damaged areas without the need for sutures. Besides, it can be used in laparoscopic surgeries without detrimental effects on the host’s immune response [177,178,179].

Although commercial anti-adhesion products based on polysaccharide materials on the market at present have shown certain advantages in terms of biocompatibility and anti-adhesion effect, they still have some shortcomings. Firstly, the high cost of these products makes it difficult for some patients to afford. Secondly, most products are only suitable for the treatment of local adhesions. In addition, certain products require specific environmental conditions to deliver optimal results, which can be challenging in harsh surgical environments. Therefore, these products still need to be further improved and perfected.

## 6. Conclusions and Future Outlook

As natural and biocompatible polymers, polysaccharide hydrogels have shown great potential in preventing postoperative adhesion. The physical and chemical properties of hydrogels can be regulated by adjusting the structure of polysaccharide materials and active substances, thus meeting the different needs of postoperative anti-adhesion. These hydrogels can effectively promote wound healing and inhibit the formation of postoperative adhesions, thereby improving patients’ quality of life. In addition, polysaccharide hydrogels are derived from diverse sources and are easy to process. However, despite extensive research into the use of polysaccharide materials for anti-adhesive hydrogels, several challenges and issues persist.

- Firstly, the hydrogels used for postoperative anti-adhesion should have an appropriate degradation rate. An excessively slow rate can lead to additional inflammation and foreign body reactions, while a rapid rate may fail to deliver the desired antiadhesion effect. And the degradation rate of pure polysaccharide materials may not be suitable for the application of anti-adhesion hydrogels.
- Secondly, hydrogels must possess adequate mechanical strength to withstand the pressures in vivo and maintain their structure; however, polysaccharide materials generally lack these mechanical properties.
- Finally, although polysaccharide materials come from a wide range of sources, the cost of their large-scale modification is high. Therefore, their large-scale production may be limited, and this may increase the financial burden on patients.

Therefore, it remains essential to further study the mechanisms of anti-adhesion, optimize the preparation process, and enhance the clinical efficacy of these hydrogels. As research progresses and technology advances, the relationship between the structures and properties of polysaccharide materials can be deeply explored. This will enable the development of polysaccharide-based hydrogels with enhanced anti-adhesion capabilities, bolstering their use in clinical settings, expanding their application in the realm of postoperative anti-adhesion, and ultimately providing patients with safer medical care.

## Figures and Tables

**Table 1:** The advantages and limitations of different polysaccharides used in hydrogels.

Polysaccharide	Structure	Advantages	Limitations
Cellulose	*β-*(1→4) linked *d*-glucose unit	Biocompatibility Biodegradability Cheap Renewability Good mechanical strength	Low solubility in water and most organic solvents Lack of antimicrobial activity
Chitosan	*β*-(1→4) linked D-glucosamine and *N*-acetyl-D-glucosamine units	Antimicrobial Antioxidant Adhesive potential Biodegradability Biocompatibility	Low solubility in water Limited mechanical strength
Alginate	1,4-β-D-mannouronic acid (M) and 1,4-α-L-gulonuronic acid (G) connected by a 1–4 glucosidic bond	Non-toxicity Biocompatibility Easy gelation Non-immunogenicity Cheap	Insufficient mechanical properties Low biological activity Slow degradation
Hyaluronic acid	*N*-acetyl-d-glucose linked by various (1,3) and (1,4) glycosidic linkages and amine and D-glucuronic acid residues	Water retention ability Biocompatibility Biodegradation The target capacity to numerous cells	High enzymatic vulnerability Weak mechanical strength
Dextran	*α*-1,6-linked glucose with 1,2, 1,3, 1,4-branch linkages	Swelling capacity Biocompatibility Biodegradability Non-toxicity	Uncontrolled hydration rate
Starch	Glucan composed of D-glucopyranose units linked by *α*-1,4- and *α*-1,6-glycosidic bonds	Biocompatibility Biodegradability Non-toxicity Low cost	Poor homogeneity Poor mechanical performance

**Table 2:** Comparison of some commercial products.

Products	Components	Target	Properties
Seprafilm^®^	Carboxymethyl cellulose Hyaluronic acid	Abdominal Pelvic cavity	Degradable Immune to blood interference Safe Inconvenient
Interceed^®^	Oxidized regenerated cellulose	Pelvic cavity	Strong adhesion Degradable Flexible Reduced effect on blood
Oxiplex/AP^®^	Carboxymethylcellulose sodium Polyethylene oxide	Uterine cavity Abdomen	Transparent May cause edema, congestion, and other problems Strong adhesion Suitable for laparoscopic surgery
BaiFeiMi	Modified chitosan	Abdomen	Good biocompatibility Rapidly forms film upon contact with bodily fluids Degradable
Nachitin	Chitosan	Abdomen	Good coagulation activity Antimicrobial Degradable Cases of conjunctivitis, fever, and other adverse reactions reported

## Data Availability

Not applicable.
